# Sleep links hippocampal propensity for epileptiform activity to its viscerosensory inputs

**DOI:** 10.3389/fnins.2025.1559529

**Published:** 2025-03-13

**Authors:** Ekaterina Levichkina, David B. Grayden, Steven Petrou, Mark J. Cook, Trichur R. Vidyasagar

**Affiliations:** ^1^Department of Optometry and Vision Sciences, The University of Melbourne, Parkville, VIC, Australia; ^2^Institute for Information Transmission Problems (Kharkevich Institute), Russian Academy of Sciences, Moscow, Russia; ^3^Department of Biomedical Engineering, The University of Melbourne, Parkville, VIC, Australia; ^4^Graeme Clark Institute, The University of Melbourne, Parkville, VIC, Australia; ^5^Florey Institute of Neuroscience & Mental Health, University of Melbourne, Parkville, VIC, Australia; ^6^Department of Medicine, University of Melbourne, Parkville, VIC, Australia; ^7^Department of Neuroscience, St. Vincent’s Hospital, University of Melbourne, Melbourne, VIC, Australia; ^8^Florey Department of Neuroscience & Mental Health, University of Melbourne, Parkville, VIC, Australia

**Keywords:** vagus, epilepsy, circadian rhythm, Hippocampus, sleep, ipRGC (intrinsically photosensitive retinal ganglion cells)

## Abstract

The development of a seizure relies on two factors. One is the existence of an overexcitable neuronal network and the other is a trigger that switches normal activity of that network into a paroxysmal state. While mechanisms of local overexcitation have been the focus of many studies, the process of triggering remains poorly understood. We suggest that, apart from the known exteroceptive sources of reflex epilepsy such as visual, auditory or olfactory signals, there is a range of interoceptive triggers, which are relevant for seizure development in Temporal Lobe Epilepsy (TLE). The hypothesis proposed here aims to explain the prevalence of epileptic activity in sleep and in drowsiness states and to provide a detailed mechanism of seizures triggered by interoceptive signals.

## Introduction

Temporal Lobe Epilepsy (TLE) is the most frequent form of focal epilepsy (60–70%) and constitutes around 24% of all cases of epilepsy (Semah et al., 1998, reviewed by Téllez-Zenteno and Hernández-Ronquillo, 2012). Among TLEs, mesial temporal lobe epilepsy (mTLE) is the most common form. It is a type of focal epilepsy originating from the medial part of the hippocampus, amygdala or the entorhinal cortex and relies on oversynchronization of activity of neuronal circuits in these areas (Diehl and Duncan, 2015). However, mTLE is likely a system disorder with network alterations due to structural and/or functional abnormalities in neocortical areas, especially the limbic, lateral temporal and frontal cortices and the thalamus (Bernhardt et al., 2013). TLE has also the highest rate of pharmaco-resistance (75–89%), only a moderate rate of successful surgical treatment at 65–70% (Sperling et al., 2005; Fanselow and Dong, 2010; Téllez-Zenteno and Hernández-Ronquillo, 2012) and an estimated Standard Mortality ratio of treatment-resistant epilepsy at 2.54 (Mohanraj et al., 2006). Although local neuronal changes associated with TLE have been extensively studied, much less is known about the triggers of seizure activity and mechanisms related to that triggering. Since frequency, severity, prodromal symptoms and patterns of occurrence across sleep–wake cycle vary between patients (Karoly et al., 2017, 2018; Lunardi et al., 2016; Niu et al., 2025), understanding the mechanisms that trigger seizures in TLE can provide substantial advantage for their prediction and treatment. Here we propose a scheme that describes a nexus between circadian rhythm, visceral inputs to the brain and the hippocampus that provides a fundamental insight into triggering of seizures in TLE and the role that stimulation of the vagus nerve can potentially fill in the management of TLE.

## Vagus nerve stimulation: an unorthodox treatment for TLE

Vagus Nerve Stimulation (VNS) is a treatment option that is being increasingly adopted for pharmaco-resistant cases of epilepsy (for recent reviews, Austelle et al., 2024; Clifford et al., 2024; Salama et al., 2024). The vagus nerve carries signals between the internal (visceral) organs, such as the lungs, heart and intra-abdominal structures and the brain. The information carried by vagal afferents is necessary for ‘interoception’, which helps the brain to process the internal signals that are related to our physiological state in both subconscious and conscious states (Craig, 2002; Quadt et al., 2018; Khalsa et al., 2018; Berntson and Khalsa, 2021; Quigley et al., 2021; Feldman et al., 2024). On average, about two thirds of patients have fewer seizures following VNS, though one third do not benefit from it (Yap et al., 2020; Elliott, 2011; Englot et al., 2011). The side effects are generally mild and often decrease over time or after optimizing the stimulation parameters individually for each patient (Sackeim et al., 2001).

The antiepileptic effects of VNS were initially attributed to widespread cortical desynchronization (Zanchetti et al., 1952), but both synchronization and desynchronization have been observed with VNS in animal studies (Chase et al., 1966, 1967). EEG studies in human patients have also reported variable outcomes (Vonck and Larsen, 2018). A hypothesis involving modulation of noradrenaline pathways (Vonck and Larsen, 2018) has also been met with criticism. The slow speed of the development of noradrenaline modulation cannot explain the immediate effects of VNS (Dorr and Debonnel, 2006), although such modulation can potentially contribute to the increase of the effectiveness of VNS over time. Another confound is the lack of VNS-associated sleep disturbances usually caused by excessive noradrenaline levels. Although some VNS parameters can provoke obstructive sleep apnoea, it has been reported to generally improve sleep architecture to the extent that VNS has been proposed as an insomnia treatment (Wu et al., 2022). The slow effects of VNS in TLE might also be related to normalization of hippocampal activity from neuronal development and growth as well as changes in sensitivity of hippocampal neurons to GABA. The latter may be because GABA_A_ receptor density in the hippocampus increases in patients responsive to VNS (Groves and Brown, 2005; Marrosu et al., 2003). VNS can also affect cytokine production, potentially controlling inflammation during long-term application (Majoie et al., 2011).

In summary, an explanation for the fast-acting component of vagal stimulation is lacking and the slow effects of VNS are also poorly understood. Thus, the method is currently used largely on an empirical basis (Yap et al., 2020; Groves and Brown, 2005). Such uncertainty means the outcome of the intervention cannot be predicted. This is an important issue, since not all patients benefit from VNS and so its effectiveness can only be established after the implantation and not prognostically (Yap et al., 2020).

## Novel model for action of VNS in TLE

We have recently proposed a new framework describing how the antiepileptic effect of VNS may arise from interruption of the resonant paroxysmal activity triggered by rhythmical interoceptive signaling (Pigarev et al., 2020). In the present paper, we discuss specifically the applicability of that hypothesis to TLE and TLE-comorbid conditions, with an emphasis on hippocampal connectivity, including signals arising from sensory systems.

**Figure 1:**
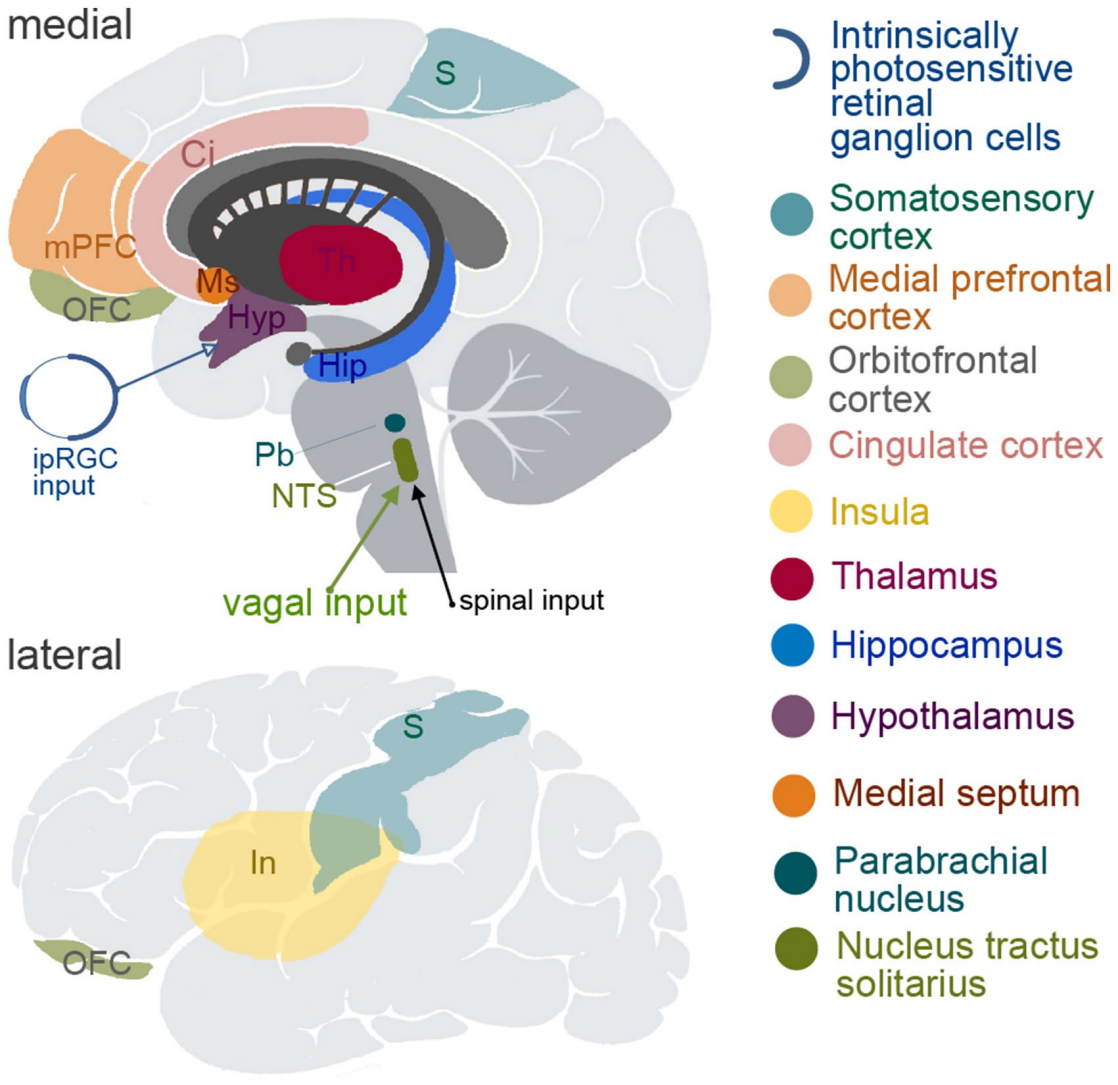
Schematic depiction of the brain structures known to be involved in viscerosensory and/or visceromotoric functions. The upper panel demonstrates medial view of the brain, and the lower panel shows lateral view. ipIGC, intrinsically photosensitive retinal ganglion cells; S, somatosensory cortical areas; mPFC, medial prefrontal cortex; OFC, orbitofrontal cortex; Ci, cingulate cortex; In, insula; Th, thalamus; Hip, hippocampus; Hyp, hypothalamus; Ms, medial septum; Pb, parabrachial nucleus; NTS, nucleus tractus solitarius.

Since approximately 80% of vagal fibers are afferent, VNS is expected to activate multiple brain areas due to the broad representation of the vagal input in subcortical and cortical structures; e.g., nucleus of the solitary tract (NTS), hippocampus, hypothalamus, thalamus and amygdala, as well as multiple cortical areas such as the insular, orbitofrontal, medial prefrontal and cingulate cortices and some somatosensory and motor cortical areas (Saper, 2002; Cechetto and Saper, 1987; Neafsey, 1990; Ongür et al., 1998; Ongür and Price, 2000; Nieuwenhuys, 2012; Azzalini et al., 2019). The most important brain regions involved in visceral sensation and regulation of visceral functions are shown in Figure 1.

### Hippocampal functional connectivity and epilepsy—in sleep and when awake

Of the many brain areas potentially influenced by VNS, the hippocampus is the most seizure-susceptible structure in the brain (Green and Scheetz, 1964) and so it warrants a close examination of its afferent and efferent projections. However, the susceptibility for seizures is not uniformly distributed across the hippocampus but increases along its dorso-ventral axis in animals (corresponding to posterior-to-anterior axis in humans), as consistently reported in kindling studies *in vitro* and *in vivo* (Bragdon et al., 1986; Elul, 1964, Strange et al., 2014; Isaeva et al., 2015; Akaike et al., 2001). There is also a matching profile of GABA_A_ receptor distribution along the same axis (Sotiriou et al., 2005). Human patients suffering from TLE also have greater structural atrophy in the anterior parts of the hippocampus in comparison to the posterior parts (Barnett et al., 2019). Furthermore, dorsal and ventral parts of the hippocampus have distinctly different connectivity with subcortical and cortical areas (Strange et al., 2014; Fanselow and Dong, 2010).

There is a specific smoothly changing pattern of connections along the above axis, which supports different goal-oriented behaviors. Dorsal hippocampus is engaged in cognitive functions including memory and spatial navigation, while ventral hippocampus is involved in emotional and affective behavior, stress-related responses and autonomic regulation. For example, lesions of dorsal hippocampus in rodents impair spatial memory (Moser et al., 1995), while ventral hippocampus lesions produce changes in emotional behavior and in reactions to stress (Kishi et al., 1990). In both rodents and primates, the dorsal hippocampus is connected with cortical and subcortical structures that form a circuit organizing exploratory and foraging activities (Swanson, 2000).

Ventral hippocampus connects extensively with structures of visceral and emotional control: amygdala, insular cortex, infralimbic and prelimbic cortices and the areas of hypothalamus that control autonomic, endocrine and somatomotor activities supporting behaviors with strong emotional components, such as feeding, reproduction and defense (Dong and Swanson, 2006; Herman et al., 2005; Kishi et al., 2000; Petrovich et al., 2001; Fanselow and Dong, 2010; Castle et al., 2005). The outermost ventral portions of hippocampal CA1 and subiculum project to hypothalamic neuroendocrine motor neurons via lateral septum and the bed *nucleus* of the stria terminalis, which is an important relay for hypothalamic–pituitary–adrenal axis of stress response (Dong and Swanson, 2006; Fanselow and Dong, 2010; Herman et al., 2016). Ventral hippocampus also projects to the shell of nucleus accumbens and shows responses related to expectation of food or to receiving food reward (Vidyasagar et al., 1991; Salzman et al., 1993). This emotion-related connectivity pattern of the ventral hippocampus may correspond to the high comorbidity of TLE and depression (Hermann et al., 2000). A recent study has highlighted both the high prevalence of depression in epilepsy and its persistence despite therapy with antidepressants (Ongchuan Martin et al., 2022). Human data regarding hippocampal dysfunction and volume loss also demonstrate association with other psychiatric conditions having an affective component, such as anxiety, bipolar and posttraumatic stress disorders (Frey et al., 2007; Bonne et al., 2008). It is notable that VNS was confirmed to be effective in treating depression and approved for use (reviewed by Nemeroff et al., 2006). This suggests the possible commonality of the VNS mechanism in epilepsy and depression.

Ventral hippocampus also possesses connections with the regions of hypothalamus that regulate the sleep–wake cycle, namely the suprachiasmatic and dorsomedial hypothalamic nuclei (Cenquizca and Swanson, 2007; Kishi et al., 2000; Krout et al., 2002; Saper et al., 2005). Interestingly, there are several factors related to sleep that could potentially play a direct role in TLE. The intrinsically photosensitive retinal ganglion cells (ipRGCs), a class of RGCs containing the photosensitive pigment, melanopsin, have a fundamental function in circadian rhythm in causing the release of melatonin in the dark through a separate pathway that does not contribute to image formation (Mure, 2021). This is via their projection to the suprachiasmatic nucleus (SCN), which in turn can send these signals on to the hippocampus through the known connections between the SCN and the medial septum (Ruby et al., 2008, 2013; Snider et al., 2018) and between the medial septum and hippocampus (Castle et al., 2005).

Intrinsically photosensitive retinal ganglion cells (ipRGCs) have their peak sensitivity in the blue part of the light spectrum, which is consistent with the seizure-preventing effects of blue light in photosensitive epilepsy (reviewed by Fisher et al., 2022). The role of the SCN was recently highlighted by Liang et al. (2024) who demonstrated that either SCN lesions or knocking out a clock gene *Bmal1* in the SCN led to an increase of seizure frequency in a mice model, as well as leading to morphological damage and alterations of GABAergic signaling in hippocampus. Core clock genes such as CLOCK, Bmal1, PER, and CRY regulate circadian rhythms, which influence the timing and occurrence of seizures in many types of epilepsy (Li et al., 2017; Castro et al., 2018; Jin et al., 2020; Kreitlow et al., 2022; Niu et al., 2025). In kainic acid-induced temporal lobe seizures, CRY1 and CLOCK were found to be dysregulated in the hippocampus (Casillas-Espinosa et al., 2023; Matos et al., 2020). The SCN, as the central circadian pacemaker, coordinates the expression of clock genes throughout the body, including in brain regions prone to seizures. This regulation modifies neuronal excitability in a time-dependent manner, potentially affecting seizure susceptibility (Gerstner et al., 2012; Chan and Liu, 2021).

Although the hippocampus has its own mechanisms of circadian regulation that influence memory functioning over a sleep–wake cycle, the suprachiasmatic nucleus is known to function as a “master clock,” providing phase setting of circadian rhythms of many structures throughout the brain (Mohawk et al., 2012), including in the hippocampus (Snider et al., 2018). This is conducted via both endocrinal and neuronal pathways. The main endocrine mechanism is the regulation of corticosterone production of the adrenal gland by the SCN and the dependence of hippocampal circadian phase on corticosterone levels. Cortisol levels have been linked to both circadian and ultradian (i.e., recurrent periods within a day, as during sleep) rhythmicities (Niu et al., 2025). The neural pathway regulating circadian phase of the hippocampus connects the SCN with hippocampus via the medial septum, as described earlier. This ultimately modulates the balance of excitation and inhibition in the hippocampus, potentially altering neural activity and synaptic plasticity (Cirelli, 2013; Wu et al., 2022). Medial septum receives inhibitory GABA-ergic signals from the SCN during subjective night phase (Ruby et al., 2008, 2013; Snider et al., 2018). In the absence of cholinergic support from the inhibited septal areas, the hippocampus switches from tonic to phasic type from its own local GABA-dependent inhibition (Wu et al., 2022). Such reduction of tonic GABA-ergic inhibition and increase of phasic inhibition is known to support an oscillatory type of neuronal activity (Farrant and Nusser, 2005), which is typical during slow wave sleep.

Notably, vagal input into the hippocampus is also transmitted via the medial septum (Castle et al., 2005; Suarez et al., 2018). This pathway thus links the major brainstem input area receiving vagal afferents, namely the nucleus of the solitary tract (NTS), to the hippocampus. We hypothesize that a confluence of signals during sleep can contribute to increased visceral oscillatory input to the hippocampus during sleep. We suggest that medial septum is a structure that provides gating of the vagal inputs based on the information coming from the SCN, increasing the strength of vagal influence during the night when it is dark and attenuating it during daytime.

Another brain area involved in regulation of hippocampal activity and in interoception is the locus coeruleus (LC), the main noradrenergic center of the brain. LC receives input from the vagus nerve via the NTS and in turn sends projections to various brain regions, including the hippocampus, amygdala, thalamus and neocortical areas and is known to be involved in processing and modulating interoceptive information (Tsetsenis et al., 2023; Fornai et al., 2011).

LC activation has been shown to modulate hippocampal synaptic plasticity. Stimulation of the LC facilitates long-term depression (LTD) in the dentate gyrus of the hippocampus, which is dependent on *β*-adrenergic receptors (Lemon et al., 2009). This LC-induced LTD may play a role in selecting salient information for subsequent synaptic processing in the hippocampus (Lemon et al., 2009). Since many interoceptive signals are of importance to the animal’s survival, they are likely to be salient on many occasions. Furthermore, VNS activates the NTS, which in turn activates LC neurons, promoting the release of noradrenaline throughout the brain including the hippocampus and the potential induction of long-term plastic changes such as those observed after prolonged VNS application (Fornai et al., 2011). LC can contribute to neuroplastic changes in the hippocampus and the control of arousal in it (Berridge and Foote, 1991). With the emerging view that epileptogenesis may involve the disturbance of normal NREM sleep-related homeostatic plasticity (Halász et al., 2019), neuroplasticity has become an important contemporary target for anti-seizure treatments (Asim et al., 2024a, b; Ma et al., 2024; Waris et al., 2024),

We have described previously increased propagation of visceral information to various cortical areas during sleep (Pigarev, 1994; Pigarev et al., 2013; Pigarev and Pigareva, 2013) and more recently we have also reported a striking increase in the responsiveness of cells in the insula to visceral stimulation during sleep (Levichkina et al., 2021) as well as a dependency of hippocampal synchronization to visceral signals on the state of vigilance (Vidyasagar et al., 2022). Gastric activity, which is communicated to the brain by vagal afferents, is also partly synchronized with the activity during resting state in many areas of the brain including the limbic system (Rebollo et al., 2018; Cao et al., 2022). It has also been recently demonstrated that shorter sleep duration in humans is associated with increased sympathetic activity, thus contributing to increased risk of hypertension and cardiovascular disease in people with shortened sleep (Tai et al., 2023). Rembado et al. (2021) demonstrated substantial increase of VNS responses during slow wave sleep (SWS) in multiple cortical areas in primates. This indicates that there are more pronounced vagal inputs during sleep in comparison to waking hours. The disturbance of such inputs caused by poor sleep might be the source of comorbidities between shortened sleep and cardiovascular diseases (Forshaw et al., 2022; Tai et al., 2023).

One cause for the increased visceral afferent signals during sleep may itself be due to increased parasympathetic stimulation of the internal organs from the vagal complex, which, in turn, receives a significant modulation from the suprachiasmatic nucleus (Ferini-Strambi and Smirne, 1997; Mutoh et al., 2003; Buijs et al., 2001; Cabiddu et al., 2012; De Zambotti et al., 2018; Karemaker, 2022). The light related changes in autonomic regulation are known to be pronounced and are mediated through the SCN, since SCN ablation aborts the autonomic responses to light (Mutoh et al., 2003). Thus, the changes in ipRGC signals from the eye in the sleep/wake cycle may be modulating the circadian signals to the vagal complex via the SCN.

As described above, there may be a number of ways in which the likelihood of seizures happening during sleep increases. If the modulation of visual signals between day and night has a significant influence on triggering TLE, it has significant implications for preventing seizures. The above-described link between visual system and epileptogenesis may also be a critical area for future investigations.

### Paroxysmal activity during sleep and TLE

The association between slow wave activity and epileptic discharges and the possibility that they may share the same mechanisms have been highlighted by Beenhakker and Huguenard (2009). There is a strong prevalence of paroxysmal activity during SWS and in drowsy states in contrast to wakefulness and REM. Roughly half of all seizures occur during SWS or drowsy states despite these states occupying less than a third of the whole sleep–wake cycle of 24 hours (e.g., Shouse et al., 1996; Herman et al., 2001; Dinner, 2002; Combi et al., 2004; Pavlova et al., 2004; Hofstra and de Weerd, 2009; Chokroverty and Nobili, 2017). Temporal lobe seizures occur more frequently during drowsiness and early stages of sleep, becoming less frequent during REM sleep (Herman et al., 2001). Furthermore, although seizures in TLE occur at equal frequency in wakefulness and sleep, as many as 75% of drug-resistant patients suffer from daytime sleepiness, reporting more frequent naps during daytime (Zanzmera et al., 2012). Thus, correct estimation of the seizure frequencies as per state of vigilance is problematic in TLE patients. Focal epilepsy seems to be provoked by sleep and sleep deprivation leads to hyperexcitability of hippocampal networks. SWS predisposes to the development of interictal epileptiform discharges in TLE and to generalization of seizure activity (for a systematic review see Garg et al., 2022). The burst-firing mode of SWS, which normally produces sleep spindles, can turn into an epileptic working mode in mTLE (Halász and Szűcs, 2020). In mTLE, there is evidence for local wake slow waves (LoWS) that share key features with slow waves in sleep (Sheybani et al., 2023). The hypersynchrony and interictal spikes present during SWS may facilitate the onset or spread of partial seizures in mTLE (Sammaritano et al., 1991; Malow et al., 1998).

TLE often shows high amplitude spikes lasting for 50–100 ms, followed by a slow wave lasting for 200–500 ms. Such events are common in SWS, when they propagate to other areas more easily due to the increased slow wave synchronization (Mendes et al., 2019). Another stereotypical activity is a “sharp wave-ripple” complex consisting of a ~ 100 ms wave followed by a high frequency ripple, initiated by synchronized activity of CA3 pyramidal neurons. The sharp wave-ripples (SPW-Rs) in the hippocampus occurring during SWS are known to be essential for memory processing and synaptic plasticity. However, an increase of the ripple frequency potentially transform the SPW-Rs into epileptic spikes, thus causing the characteristic “fast ripples” in epilepsy (Staba et al., 2007; Maier and Kempter, 2017). Recent studies have demonstrated the mechanism of the natural ripple control during sleep. It involves cholecystokinin-expressing basket cells in the hippocampus (Karaba et al., 2024; Amin, 2024). Cholecystokinin (CCK) is known to increase neuronal activity in the hippocampus (Asim et al., 2024a). Furthermore, in the hippocampus, CCK immunoreactivity has been found to coexist with GABA (Somogyi et al., 1984) and CCK-induced hypersensitivity in the auditory system has been linked to sound-triggered seizures (Fedotova et al., 2021). CCK receptors have extensive presence in the brain, including hippocampus, amygdala and the cortex. They modulate both GABA and glutamatergic systems and affect paroxysmal activity (Asim et al., 2024a, b). CCK enhances the inhibitory tone provided by hippocampal GABAergic neurons by increasing GABA release (Miller et al., 1997). Such modulation of GABAergic activity by CCK may contribute to sleep regulation and hippocampal slow wave activity, as GABAergic neurons play a critical role in promoting SWS (Saper and Fuller, 2017) and in regulation of hippocampal oscillatory behavior (Földy et al., 2007; Whissell et al., 2015; Klausberger et al., 2004).

Synchronous activity of hippocampal CA3 cells is also inhibited by adenosine, which builds up during waking hours and whose levels drop by at least 20% during SWS in comparison to wakefulness (Basheer et al., 2004). This increases the probability of CA3 neurons to ovesynchronize during SWS, since both ripples and fast ripples occur mostly during sleep.

### Visceral triggers of hippocampal paroxysmal activity

The question now arises as to what may facilitate the slow wave oversynchronization that is expected to drive susceptible ventral hippocampal circuits into paroxysmal activity. A highly plausible candidate that we propose is the afferent rhythmic signaling coming from visceral systems. Breathing, heart rate, gastric and intestinal activities are all rhythmic. Hippocampal breathing rhythm is a particularly well-known phenomenon and the hippocampal sharp wave-ripple complex can be entrained by respiration (Liu et al., 2017; Lockmann et al., 2016; Radna and MacLean, 1981; Bordoni et al., 2018). This breathing-associated rhythmic activity is also present in prefrontal cortex, especially in areas connected with the olfactory system. This provides a ground for synchronization of large brain networks by breathing due to their extensive connections to the hippocampus. Frequency of the hippocampal breathing rhythm is different in different species, with the one reported in human epileptic patients being around 0.16–0.33 Hz (Zelano et al., 2016) while, in smaller animals, this rhythm is faster and can be mistaken for delta or theta activity (Lockmann et al., 2016). In a study on the relationship between visceral events such as heart rate and breathing with hippocampal cell responses in human patients, a large proportion of neurons in hippocampus and amygdala were found to either synchronize with the heart rate itself (20%) or respond to changes in heart rate (23%), in addition to 15% of cells being synchronized to the respiratory period (Frysinger and Harper, 1989). Neuronal responses associated with cardiac rhythm were studied in human subjects by Kim et al. (2019), who confirmed their presence during the resting state in hippocampus, parahippocampal cortical areas, amygdala, and the cingulate cortex.

Our framework is consistent with the many studies that have reported comorbidities between epilepsy and cardiac and respiratory illnesses (e.g., Doherty et al., 2022; Gaitatzis et al., 2004). Cardiovascular disorders which can potentially cause abnormal rhythms such as atrial fibrillation and myocardial infarction show significant comorbidity with epilepsy (Doherty et al., 2022). Respiratory rhythm is of particular interest due to its connection to Sudden Unexpected Death in Epilepsy (SUDEP), which predominantly occurs in sleep (Purnell et al., 2018; Niu et al., 2025). There is a strong association between epilepsy and sleep-related respiratory conditions such as obstructive and central sleep apnoeae. Obstructive sleep apnoea (OSA) occurs in 10% of adult epilepsy patients, 20% of children with epilepsy and up to 30% of drug-resistant epilepsy cases (Manni and Terzaghi, 2010; Zanzmera et al., 2012; Sivathamboo et al., 2019), while treatment with continuous positive airway pressure (CPAP) has been shown to improve seizure control in some cases (Malow et al., 2008). Patients with TLE have been reported to have a higher risk of obstructive sleep apnoea as well (Yildiz et al., 2015). However, the most severe comorbidity exists between TLE and central sleep apnoea, particularly ictal central apnoea (ICA). ICA has a higher prevalence in TLE compared to extratemporal epilepsy. It was observed in 36.9% of seizures and 43.2% of patients with focal epilepsy, all of whom had temporal lobe involvement (Lacuey et al., 2018; Lacuey et al., 2019; Vilella et al., 2019). In a study of patients with mTLE, the incidence of ICA was reported to be as high as 68.7% (Vilella et al., 2019). Thus, the association between TLE and central apnoea appears to be often related to the involvement of temporal lobe structures in respiratory control. There is also comorbidity between bronchial asthma and epilepsy. However, it varies across studies, ranging between 9 and 13.4% (Gaitatzis et al., 2004; Téllez-Zenteno et al., 2005; Chiang et al., 2018; Doherty et al., 2022). It is also interesting that, at 22%, the condition having the highest comorbidity with epilepsy is the group of anxiety disorders (Doherty et al., 2022). A typical and common symptom in anxiety disorders is hyperventilation. It is notable that hyperventilation, used as an activation method to provoke interictal discharges and seizures during video-EEG monitoring of epilepsy patients is more effective in temporal lobe patients (Guaranha et al., 2005).

When the activity in those with TLE falls roughly into the delta frequency range and seems to be correlated with cardiac activity, it is possible that oversynchronisation may lead to epileptic seizures. However, this raises a few issues: (i) In smaller animals, which usually tend to have faster heart rates, higher frequency ranges would be expected to become oversynchronised, which may not be the case. (ii) In the over 50% of the cases where no correlation with cardiac or breathing rhythm is seen, is there another rhythmic activity that could provide the trigger for seizures?

The above leads to the possibility that rhythmic signals from abdominal viscera may set off an over-synchronization. Hippocampus is not only known to receive vagal inputs as described earlier, but it is also engaged in organization of feeding behavior and has been shown to receive signals from the gastrointestinal tract, with neurons in the macaque hippocampus and parahippocampus showing responses related to food reward (Vidyasagar et al., 1991; Tamura et al., 1991; Salzman et al., 1993). Positron Emission Tomography (PET) studies in humans have also revealed hippocampal responses to gastric stimulation (Wang et al., 2006). We have recently reported a coupling between gastrointestinal myoelectric activity and the spiking of hippocampal cells (Vidyasagar et al., 2022). Furthermore, it was shown that vagal afferents that transmit information from the stomach to the brain may act as a peripheral clock themselves and that mechanosensitivity of these vagal afferents changes over a 24-hour period, indicating that their responses to mechanical stimuli are not static but exhibit circadian variation (Kentish et al., 2013).

Serotonin and gut peptides such as CCK, peptide-YY and leptin released in response to gastric distention provoke satiety and somnolence via activation of the vagus nerve (Kim and Lee, 2009). Thus, though CCK does not cross the blood–brain barrier, it affects neural activity in the brain via the vagus nerve (Cao et al., 2012). Furthermore, capillary endothelial cells of the blood–brain barrier expressing CCK-1 receptors change their permeability to other molecules including leptin in response to CCK (Cano et al., 2008). Leptin plays a significant role in hippocampal neuroplasticity, affecting multiple molecular pathways involved in synaptic changes (Li et al., 2002; Moult and Harvey, 2011; Dhar et al., 2014; McGregor et al., 2018). It facilitates LTP in the CA1 region and the dentate gyrus, which are believed to underlie learning and memory formation (Shanley et al., 2001; Oomura et al., 2006). It also influences excitatory synaptic transmission in the hippocampus. In adult tissue, it induces a persistent increase in excitatory synaptic transmission, termed leptin-induced LTP (Li et al., 2002). Interestingly, intraperitoneal CCK injections led to increase of EEG slow wave activity and sleep (Kapás et al., 1988). The above studies indicate a strong association between increased plasma CCK levels and TLE (Gao and Bao, 2020).

Thus, there are multiple ways in which gastrointestinal events can potentially modulate hippocampal activity and there is reason to believe that rhythmic gastrointestinal events have different patterns in different states of vigilance. Some of these patterns could also lead to rhythmic activity in the hippocampus, potentially triggering seizures. We propose that all the variants of TLE, including those where correlation or modulation with easily recordable visceral events such as breathing or heart rate are not observed, share essentially the same mechanism of being susceptible to be triggered by interoceptive slow waves. We suggest that networks susceptible to paroxysmal activity that include the ventral hippocampus may be triggered by any of the afferent visceral signals, including those arising from the gastrointestinal tract.

Autonomic disturbances accompany TLE seizures in up to 75% of cases and are frequent during the aura period (van Buren and Ajmone-Marsan, 1960). Ictal autonomic changes include cardiorespiratory, gastrointestinal, vascular, urogenital and pupillary symptoms (Dütsch et al., 2006). The most commonly reported seizure triggers relate to particular visceral states such as menstruation, sleep deprivation, fatigue, eating, fever, et cetera or to stress and anxiety (Lunardi et al., 2011, 2016), indicating that such changes do not just accompany, but may also induce paroxysmal activity.

### External modulation of visceral triggers of hippocampal paroxysmal activity

Externally driven seizure events are not uncommon and reflex epilepsy is a well-known example of the epileptiform activity triggered by sensory stimuli. Seizures in TLE can be provoked by rhythmic olfactory stimulation (Lunardi et al., 2016), mediated by strong projections from the olfactory system to hippocampus. Olfactory auras also occur in drowsy/dreaming states in 6% of TLE patients. TLE-associated, ‘eating epilepsy’ has been described as well (Nagaraja and Chand, 1984). Here, sight, smell or thought of food did not cause seizures and they only rarely ever occurred during eating itself. In most cases, the seizures occurred at the end of a heavy meal, suggestive of the higher gastrointestinal activity associated with digestion as the trigger and partial control of seizures was achieved simply by changing the eating habits. Another example of epileptic activity associated with gastrointestinal function is the seizure resulting from gastroenteritis in children (Cusmai et al., 2010). Irritable bowel syndrome also increases the risk to develop epilepsy (Chen et al., 2015). Our proposal provides a neural framework for these phenomena.

**Figure 2:**
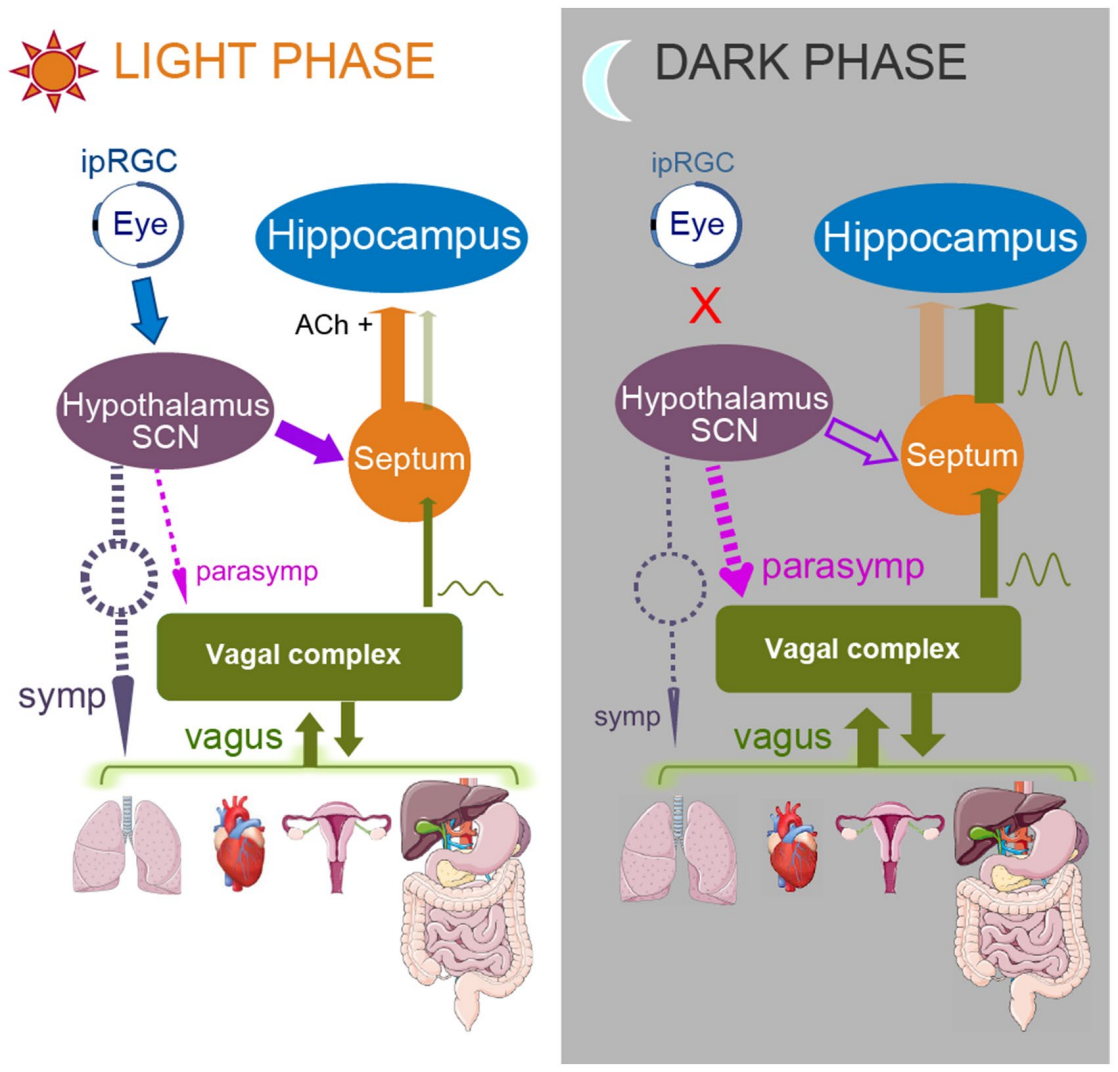
Model of functional connectivity changes occurring upon transition from light to dark phase of a sleep–wake cycle. Left panel represents subjective light phase, and right panel represents subjective dark phase. During the light phase, ipRGCs of the retina are activated by light and send their signals to SCN. SCN stops producing melatonin, thus enhancing sympathetic activity (dashed gray arrow). As a result, predominantly parasympathetic vagal activity (dashed purple and green arrows) is attenuated. SCN also does not inhibit medial septum during the light phase; that allows disinhibited septal areas to send cholinergic signals to the hippocampus (orange arrow, Ach+), promoting arousal and supporting tonic type of hippocampal inhibition. Medial septum also “closes the gate” for the vagal signals, reducing chances for hippocampal triggering by the vagal input. In contrast, during the dark phase, when SCN does not receive ipRGC input, it increases melatonin production, supporting parasympathetic activity (dashed purple arrows) and so the vagal activity is enhanced. SCN also inhibits medial septum (open purple arrow), which decreases the medial septum’s cholinergic input to the hippocampus, causing switching of the hippocampus to a phasic inhibition mode with increased susceptibility to low frequency entrainment. Medial septum also “opens the gate” for the vagal signals, thus increasing the influence of the visceral activity on hippocampus and allowing the triggering of paroxysmal responses to occur.

Figure 2 summarizes our model of changes of functional connectivity that predispose to facilitation of seizures originating in the ventral hippocampus during the dark (night) phase of the sleep–wake cycle (right panel) in comparison to their relative suppression during the light (day) phase (left panel). During the light phase, signals from the ipRGC stimulate neurons in the SCN, which leads to reduction of the SCN suppression of the medial septum, which is known to project to the hippocampus (Ruby et al., 2008; Snider et al., 2018). The medial septum supplies cholinergic support to hippocampus and also causes hippocampal cells to receive inhibition in a tonic mode (Kalsbeek et al., 2006; Wu et al., 2022). However, during the night, in the absence of light, ipRGCs stop sending their signals to SCN, which suppresses medial septum via GABA-ergic connections. Thus, during night, there is a decrease in the cholinergic input from the medial septum to hippocampus and as a result, hippocampal cells are likely to switch to the phasic inhibition mode (Wu et al., 2022). We hypothesize that the vagal input which comes to the medial septum from NTS is also disinhibited during the night. In addition, vagal and general parasympathetic activity are more pronounced during slow wave, non-rapid eye movement (NREM) sleep (Trinder et al., 2001; Cabiddu et al., 2012; De Zambotti et al., 2018). Thus, during slow wave sleep, enhanced and potentially disinhibited vagal signals have higher chances of influencing ventral hippocampus. The other structures receiving vagal input can also be vulnerable to seizures triggered by interoceptive signals, especially the insular, anterior cingulate, orbitofrontal, somatosensory and motor cortices. One particularly striking example is frontal lobe epileptic seizures that consistently show predisposition to occur during sleep (Herman et al., 2001). Furthermore, a strong association exists between frontal lobe seizures and NREM sleep. Nobili et al. (2012) reported that in patients with nocturnal frontal lobe epilepsy (NFLE), 98% of all seizures occurred in NREM sleep. Of these NREM seizures, 72% emerged from slow wave sleep (SWS). Many frontal lobe seizures also originate from the primary motor cortex or the orbitofrontal cortex (Wang et al., 2019; Herman et al., 2001), which are areas that receive vagal inputs.

However, the hippocampal network is especially vulnerable to seizures due to multiple factors such as heightened risk of sclerosis, high density of glutamate receptors that increases chances of excitotoxicity and neuronal hyperexcitability, enhanced blood–brain barrier permeability compared to other brain regions, which makes it more susceptible to circulating toxins and pathogens causing inflammation, increased oxygen demand leading to higher sensitivity to hypoperfusion and low oxygen saturation (Sloviter, 1987; Olney et al., 1986; Marchi et al., 2007; Wei et al., 2014; Montagne et al., 2015; Mitra et al., 2016).

### Mechanism of visceral trigger of TLE seizures

There is a case for arguing that slow waves such as from the viscera (heart and lungs and the gastrointestinal tract) can induce seizure activity which is usually at higher frequencies. Slowing of EEG activity with irregular delta waves occurs in over 60% of TLE patients (Jan et al., 2010). Stimulation frequencies in a low range, such as 1 Hz, are used for pre-operative testing aimed to provoke epileptic seizures to define their source. This stimulation induces seizures when applied to the hippocampus and related structures of the hippocampal gyrus and propagate in ways that match seizure propagation (Corcoran and Cain, 1980; Munari et al., 1993). It seems likely that natural visceral activity occurring at comparable low frequencies, such as cardiac rhythms, can also initiate the same processes through resonance.

The frequency range of oscillatory activities, both normal and ictal, is determined by the biophysical properties of the morphological cell types of the oscillating cell assembly and the balance of excitation and inhibition on the cells of the assembly (Hutcheon and Yarom, 2000; Economo and White, 2012; Markram et al., 2004). These cell assemblies, by virtue of the circuitry they are embedded in, would also have a resonant frequency to which they are most susceptible (Hutcheon and Yarom, 2000; Herrmann, 2001). This has been pointed out in the general context of communication through coherence between brain regions (Vidyasagar, 2013; Esghaei et al., 2022) and the concept is extended more specifically in its potential to trigger ictal activity (Pigarev et al., 2020; Sohanian Haghighi and Markazi, 2019). Thus, when there is sufficient overlap between the frequency ranges of the interoceptive wave and the local hippocampal oscillatory activity to trigger the resonant frequency in the hippocampal circuit, a typical ictal over-synchronization can potentially occur. Such overlap and over-synchronization at the resonant frequency of the hippocampal circuit may be the result of a widening of the frequency bandwidth of one or both circuits, thanks to an abnormal hippocampal circuit from a lesion and/or a faster than normal visceral oscillation. Consistent with this idea, dependence of stimulation treatment outcomes on its frequency, with low frequencies being pro-convulsive while high frequencies being anti-convulsive, has been demonstrated for both deep brain stimulation and spinal stimulation approaches to epilepsy (Waris et al., 2024).

However, it still remains to be explained how the slow interoceptive waves (mostly <1 Hz, e.g., breathing or gastrointestinal activity) lead to the ictal over-synchronization that occur at higher frequencies and the spread of the seizure from the hippocampus to various cortical areas. For example, patients with TLE often have “sinusoidal” patches of ictal activity at frequencies of 5–10 Hz (Jan et al., 2010), and fast ripples occurring at very high frequencies (>150 Hz) are characteristic of hippocampal epileptiform activity (Foffani et al., 2007; Ogren et al., 2009).

One possibility is the occurrence of resonance at harmonics of the fundamental frequency, as was pointed out earlier (Pigarev et al., 2020). Thus, in a study of photosensitive epilepsy when photic stimulation was applied at 10–20 Hz, synchronized activity at harmonically related frequencies occurred within 30–120 Hz (Parra et al., 2003). The other option has been recently described by Stark et al. (2022). In their experiment, hippocampal pyramidal cells in CA1 were optically stimulated at 5 Hz, which resulted in slow depolarization and oscillatory response at frequencies between 60 and 80 Hz. This was followed by application of linear chirp stimulation (sinusoidal waveform stimulation with frequency changing over time linearly from 0 to 100 Hz), which revealed a resonant peak of a cell to occur at 40 Hz. Thus, high frequency oscillations can result from stimulation well outside of the cell’s resonant peak, and a neuronal oscillator does not have to be a resonator to show evoked oscillations.

**Figure 3:**
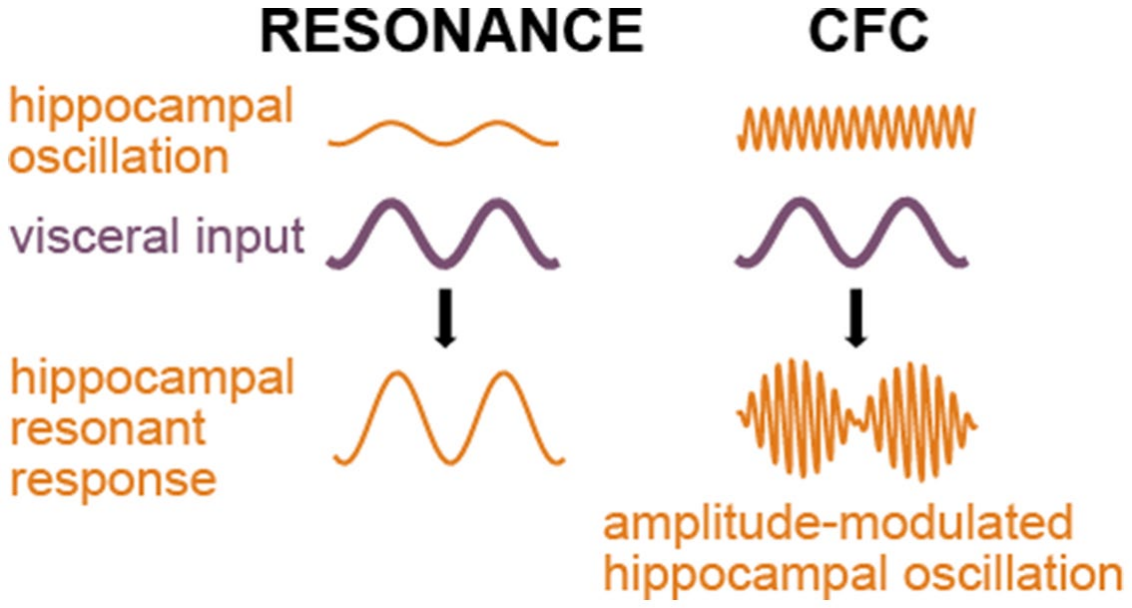
Outcomes of the resonant and phase-amplitude CFC mechanisms of seizure triggering. Both mechanisms require visceral input (purple); however, the resonance mechanism (left side of the picture) implies similarity between frequencies of the hippocampal oscillation and the visceral input or a harmonic relationship between them, while phase-amplitude CFC (right side) leads to an amplitude modulation of any prevailing higher-frequency hippocampal activity.

Yet another and likely a more common mechanism is cross-frequency coupling, which has been suggested as an efficient means of communication between brain areas (Canolty and Knight, 2010; Lisman and Jensen, 2013; Vidyasagar and Levichkina, 2019; Esghaei et al., 2022). CFC can be either amplitude-amplitude coupling, when the two frequencies are similar and oscillation in area A directly adds to the oscillation in its target area B, or phase-amplitude coupling, when the phase of lower frequency oscillation in area A modulates the amplitude of a local higher frequency oscillation in area B. Normal hippocampal ripples are known to be entrained by breathing (Liu et al., 2017; Lockmann et al., 2016; Radna and MacLean, 1981; Bordoni et al., 2018; Nokia and Penttonen, 2022). As pathological fast ripples in the hippocampus have similar origin as the normal ripples (Foffani et al., 2007), it seems safe to assume that CFC-based entrainment of respiration or other low frequency visceral events can cause fast ripples as well. Figure 3 schematically compares the influence of two potential mechanisms of seizure-triggering on the ongoing hippocampal oscillations.

That the visceral waves usually have a frequency range that barely overlaps with the theta, beta and gamma frequencies typically seen in most of the brain regions, including the hippocampus, explains why seizures are not easily triggered, at least during waking hours when the vagal afferent activity from gastrointestinal tract is less (Pigarev, 1994; Pigarev et al., 2013; Rembado et al., 2021; Levichkina et al., 2021; Levichkina et al., 2022). However, we suggest that, with an altered abnormal hippocampal circuit, this protection is lost, especially during sleep, when the visceral oscillations are stronger.

The next question to address is the spread of activity from the hippocampus to other brain regions, such as the frontal cortex, which receives a strong projection from the hippocampus (Barbas and Blatt, 1995; Thierry et al., 2000; Catenoix et al., 2011), sometimes leading to generalized convulsions. CFC-mediated spread of hippocampal seizure may be further augmented by the claustrum, consistent with the proposed function of the claustrum with a unique morphology and connectivity that enables it to enhance neural synchrony between brain areas (Vidyasagar and Levichkina, 2019; Madden et al., 2022). The claustral involvement is also consistent with the finding that during sleep, the slow wave activity is enhanced through claustral projections to widespread neocortical areas (Narikiyo et al., 2020). It has been pointed out that the claustral projections are uniquely organized to rapidly abort the enhanced neural synchrony between cortical areas (Vidyasagar and Levichkina, 2019). This could potentially explain why such augmentation of synchrony does not usually cause seizures and why claustral damage, on the other hand, can sometimes cause seizures (Meletti et al., 2017).

## Implications for therapy

The above account of the role of the vagus in transmitting the visceral signals to the brain, including the hippocampus, provides a basis for the efficacy of vagal nerve stimulation (VNS) in controlling seizures in many cases of pharmacoresistant epilepsy. VNS stimulation can potentially desynchronise the hippocampal network that is causing, or about to cause, a seizure (Pigarev et al., 2020). In that case, there is no need for VNS to desynchronize the whole cortex to be effective. In fact, consistent with our framework, studies show that patients who respond favorably to VNS in comparison to non-responders exhibit more heterogeneity in the brain areas where desynchronisation in the theta band EEG is most pronounced (Vespa et al., 2021; Clifford et al., 2024). Furthermore, stronger desynchronisation in the theta band during sleep compared to wakefulness was found to distinguish VNS responders from non-responders (Vespa et al., 2021).

From the above point of view, we suggest both VNS and ventral hippocampus stimulation may be effective in seizure prevention. However, VNS implantation has less risk and is, therefore, may be preferable in many cases.

In conclusion, we propose that TLE is largely a reflex type of epilepsy with visceral and external events serving as triggers. These triggers are expected to be more effective in provoking resonant activity and over-synchronization in the hippocampus in sleep and during darker hours. From that point of view, effectiveness of VNS relies on its ability to stop such over-synchronization of the networks involved in the analysis of visceral information.

## Data Availability

The original contributions presented in the study are included in the article. Further inquiries can be directed to the corresponding authors.
